# MapReduce Based Personalized Locality Sensitive Hashing for Similarity Joins on Large Scale Data

**DOI:** 10.1155/2015/217216

**Published:** 2015-04-30

**Authors:** Jingjing Wang, Chen Lin

**Affiliations:** ^1^School of Information Science and Technology, Xiamen University, Xiamen 361005, China; ^2^Shenzhen Research Institute of Xiamen University, Shenzhen 518058, China

## Abstract

Locality Sensitive Hashing (LSH) has been proposed as an efficient technique for similarity joins for high dimensional data. The efficiency and approximation rate of LSH depend on the number of generated false positive instances and false negative instances. In many domains, reducing the number of false positives is crucial. Furthermore, in some application scenarios, balancing false positives and false negatives is favored. To address these problems, in this paper we propose Personalized Locality Sensitive Hashing (PLSH), where a new banding scheme is embedded to tailor the number of false positives, false negatives, and the sum of both. PLSH is implemented in parallel using MapReduce framework to deal with similarity joins on large scale data. Experimental studies on real and simulated data verify the efficiency and effectiveness of our proposed PLSH technique, compared with state-of-the-art methods.

## 1. Introduction

A fundamental problem in data mining is to detect similar items. Finding similar pairs of instances is an essential component in mining numerous types of data, including document clustering [1, 2], plagiarism detection [3], image search [4], and recommender system [5].

Identifying pairs of similar instances is also called similarity joins [6]. Given a set of data instances, a similarity threshold *J*, and a join attribute *a*, the goal of similarity joins is to find all pairs of instances 〈*A*, *B*〉 where their similarity on the join attribute is larger than the threshold *J* (i.e., sim(*A*.*a*, *B*.*a*) ≥ *J*). There are various similarity measurements, including cosine similarity [7], edit distance [6, 8, 9], hamming distance [7, 10], dimension root similarity [2], and EDU-based similarity for elementary discourse units [11]. In this work we focus on Jaccard similarity, which is proven to be successful for high dimensional, sparse feature sets [6]. Jaccard similarity for two feature vectors *S*_1_ and *S*_2_ is defined as sim(*S*_1_, *S*_2_) = |*S*_1_∩*S*_2_|/|*S*_1_ ∪ *S*_2_|. As an example, we illustrate naive computation for similarity joins based on Jaccard similarity in Table 1. Suppose there are 5 instances, namely, *A*, *B*, *C*, *D*, and *E*, the join attribute consists of features *a*, *b*, *c*, *d*, *e*, *f*, the Jaccard similarity of 〈*A*, *B*〉 is 1/4, 〈*A*, *C*〉 is 2/3, 〈*B*, *C*〉 is 1/5, 〈*B*, *E*〉, 〈*C*, *D*〉, and 〈*C*, *E*〉 is 1/4, 〈*D*, *E*〉 is 1/3, and the similarities for remaining pairs 〈*A*, *D*〉, 〈*A*, *E*〉, 〈*B*, *D*〉 are all 0. Given the similarity threshold *J* = 0.5, it is directly concluded that instances *A* and *C* are similar.

A naive algorithm, which finds similar pairs by computing similarities for all instance pairs, is clearly impracticable on a large collection of instances with high dimensional features. To improve efficiency and scalability of similarity joins, previous research efforts generally fall into two categories. On one hand, parallel algorithms are adopted on clusters of machines. Most of them are implemented using MapReduce framework, including a 3-stage MapReduce approach for end-to-end set-similarity join algorithm [12], fast computation of inner products for large scale news articles [13], and a new ant colony optimization algorithm parallelized using MapReduce [14] to select features in a high dimension space. Others exploit the parallelism and high data throughput of GPU, that is, the LSS algorithm [15]. On the other hand, algorithmic design can be improved to reduce time and storage cost of similarity computation for high dimensional feature space. One type of such approaches uses dimension reduction technologies, including Principle Components Analysis and neural networks [16]. Another type is to hash and filter, so that high dimensional feature space can be replaced by smaller representative signatures. Most popular hashing methods include minhashing [17], minwise hashing [18], and Locality Sensitive Hashing (LSH) [10]. The core idea of hashing is to map similar pairs to similar signatures with several hundred dimensions, each element of which is the result of hashing and hence sheds insights to the solution of high dimensionality. Hashing can also be a means for data clustering because it enables similar features with vast dimensions to be hashed into the same buckets and thus partitions features into groups [19]. Filtering methods, including length filter [7], prefix filter [20], and suffix filter [21], are frequently utilized consequently to eliminate dissimilar pairs while possible similar pairs remain. As a result, fewer similarity computations are needed. In particular, banding technique [22], a specified form of Locality Sensitive Hashing, which maps every band of signatures to an array of buckets so the probability of collision is much higher for instances close to each other, is the most efficient filtering method.

Although previous works have demonstrated the importance and feasibility of hashing and filtering approaches, one critical issue remains underestimated. Hashing and filtering approaches produce approximate results. The similarities of selected pairs are not guaranteed to be larger than the predefined threshold. In the meanwhile, obsoleted pairs are not indeed dissimilar, with similarities less than the predefined threshold. The former case is called false positive, while the latter one is called false negative. An appropriate number of false positives and false negatives are acceptable in many applications. However, the tolerance to false positive and false negative may differ. In most application scenarios such as clustering and information retrieval, a small amount of false positives is emphasized to increase efficiency and precision. In applications such as recommendation and bioinformatics systems [23–25], a small number of false negatives are more important.

In this paper, we address the problem of tailoring the number of false positives and false negatives for different applications. To the best of our knowledge, this is the first time in literature to present such detailed analysis. False positives and false negatives are caused by the scheme of pruning candidate pairs whose signatures map into disjoint bucket arrays. Intuitively, similar signatures are likely to have highly analogous bands. And analogous bands will be mapped into identical bucket arrays. Inspired by this intuition, we propose the new banding technique called Personalized Locality Sensitive Hashing (PLSH), in which bands of signatures mapped to at least *k* identical buckets are selected as candidates. We also explore the probability guarantee of the new banding techniques provided for three cases, namely, false negatives, false positives, and the sum of both. According to these probabilities, we propose the upper bounds and lower bounds of false positives and false negatives and accordingly present to personalize the parameters involved in banding and hashing algorithms to fulfill different application demands.

The contributions of this paper are threefold:

- We improve the traditional banding technique by a new banding technique with flexible threshold to reduce the number of false positives and improve efficiency.
- We derive the number and lower/upper bound of false negatives and false positives and balancing between them for our new banding technique.
- We implement the new banding technique using parallel framework MapReduce.

The rest of the paper is structured as follows. In Section 2, the backgrounds of minhashing and banding technique are presented. In Section 3, we introduce Personalized Locality Sensitive Hashing (PLSH). The implementation of PLSH using MapReduce is shown in Section 4. In Section 5, we present and analyze the experimental results. We survey the related works in Section 6. Finally, the conclusion is given in Section 7.

## 2. Background

In this section, we briefly introduce the minhashing algorithm and the consequent banding algorithm, which are the fundamental blocks of Locality Sensitive Hashing (LSH). The intuition of minhashing is to generate low dimensional signatures to represent high dimensional features. The intuition of banding is to filter candidates which are not likely to be similar pairs.

### 2.1. MinHashing

For large scale data sets, feature space is usually high dimensional and very sparse; that is, only a tiny portion of features appear in a single instance. In order to reduce the memory used to store sparse vector, we use a signature, an integer vector consisting of up to several hundred elements to represent an instance. To generate a signature, we first randomly change the order of features. In other words, the permutation defines a hash function *h*_*i*_ that shuffles the features. Each element of signature is a minhash value [17], which is the position of the first nonzero feature in the permuted feature vector. For example, the original feature vector in Table 1 is *abcdef*; suppose the permuted feature vector is *bacdfe*; then feature vectors for *A*, *B*, *C*, *D*, and *E* become (100001), (010011), (100101), (001100), and (000110) as illustrated in Table 2. Thus the minhash value for *A*, *B*, *C*, *D*, and *E* is 1, 2, 1, 3, and 4, respectively.

We can choose *n* independent permutations *h*_1_, *h*_2_,…, *h*_*n*_. Suppose the minhash value of an instance *S*_*i*_ for a certain permutation *h*_*j*_ is denoted by min⁡⁡*h*_*j*_(*S*_*i*_); then the signature denoted by Sig(*S*_*i*_) is

(1) $$\begin{array}{c} \text{Sig}\left(S_{i}\right)=\left(\min h_{1}\left(S_{i}\right),\min h_{2}\left(S_{i}\right),\ldots ,\min h_{n}\left(S_{i}\right)\right). \end{array}$$

The approximate similarity between two instances based on their signatures is defined as the percentage of identical values at the same position in the corresponding signatures. For example, given *n* = 6, Sig(*S*_1_) = (2,1, 5,0, 3,2), and Sig(*S*_2_) = (2,1, 3,2, 8,0), the approximate Jaccard similarity is sim(*S*_1_, *S*_2_) ≈ 2/6 = 0.33.

### 2.2. Banding

Given a large set of signatures generated in Section 2.1, it is still too costly to compare similarities for all signature pairs. Therefore, a banding technique is presented consequently to filter dissimilar pairs.

The banding technique divides each signature into *b* bands, where each band consists of  *r* elements. For each band of every signature, the banding technique maps the vector of *r* elements to a bucket array.

**Figure 1:**
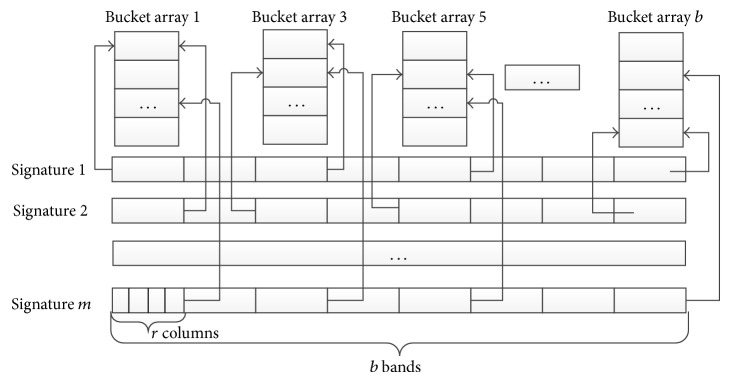
An illustrative example of banding technique.

As shown in Figure 1, the *i*th band of each signature maps to bucket array *i*. Intuitively, if for a pair of signatures, the corresponding bucket arrays have at least one bucket array in common, then the pair is likely to be similar. For example, signature 1 and signature 2 and signature 2 and signature *m* in Figure 1 are similar. Such a pair with common bucket array is considered to be a candidate pair and needs to be verified in the banding technique.

## 3. Personalized LSH

### 3.1. New Banding Technique

The candidates generated by LSH are not guaranteed to be similar pairs. Chances are that a pair of signatures are projected to identical bucket arrays even if the Jaccard similarity between the pair of instances is not larger than the given threshold. In the meantime, a pair of instances can be filtered out from candidates since their corresponding signatures are projected into disjoint bucket arrays even if the Jaccard similarity is smaller than the given threshold. The former case is called false positive, while the latter one is called false negative. Massive false positives will lead to inaccurate results, while a large amount of false negatives will deteriorate computational efficiency of LSH. To enhance the algorithm precision and efficiency, we present here a new banding scheme to filter more dissimilar instance pairs. Intuitively, if two instances are highly alike, it is possible that many bands of the two corresponding signatures are mapped to identical buckets. For example, in Figure 1, there are at least 3 bands (i.e., the 1st, the 5th, and the *b*th bands) of signature 1 and signature 2 which map to the same buckets (i.e., in the corresponding bucket array 1, 5, *b*).

Therefore, we change the banding scheme as follows. For any pair of instances, if the two corresponding signatures do not map into at least *k*  (*k* ∈ [1, *b*]) identical buckets, it will be filtered out. Otherwise, it is considered to be a candidate pair and the exact Jaccard similarity is computed and verified. For the signatures shown in Figure 1, given *k* = 3, signature 1 and signature *m* and signature 2 and signature *m* are filtered.

### 3.2. Number of False Positives

A candidate pair 〈*S*_1_, *S*_2_〉 is false positive, if sim(〈*S*_1_, *S*_2_〉) < *J* and *S*_1_, *S*_2_ share at least *k* common bucket arrays. Since the efficiency of LSH is mainly dependent on the number of false positives, and most real applications demand a high precision, we first derive the possible number of false positives generated by the new banding technique.

**Lemma 1 .**

The upper bound of false positives generated by the new banding technique is equal to the original LSH and the lower bound is approximate to 0.

**Proof**

According to the law of large numbers, the probability that the minhash values of two feature vectors (e.g., *S*_1_, *S*_2_) are equal under any random permutation *h*, is very close to the frequency percentage of observing identical value in the same position at two long signatures of the corresponding feature vectors. That is,

(2) P?min⁡hS1=min⁡hS2 =limn→+∞?SigS1r=SigS2rSigS1,

where *n* is the length of signatures Sig(*S*_1_) and Sig(*S*_2_); *r* is the position in signatures, *r* ∈ [1, *n*].

Also, the probability that a random permutation of two feature vectors produces the same minhash value equals the Jaccard similarity of those instances [17]. That is,

(3) $$\begin{array}{c} P?\left(\min h\left(S_{1}\right)=\min h\left(S_{2}\right)\right)=\frac{\left|S_{1}\cap S_{2}\right|}{\left|S_{1}\cup S_{2}\right|}=\text{sim}\left(S_{1},S_{2}\right). \end{array}$$

Based on the above two equations, the probability of two instances with Jaccard similarity *s* is considered to be a candidate pair by the new banding technique denoted by *P*_new_ as

(4) $$\begin{array}{c} P_{\text{new}}\left(s\right)=1-\;\overset{k-1}{\underset{i=0}{\sum }}\left(\begin{array}{c} i\\ b \end{array}\right){\left(s^{r}\right)}^{i}{\left(1-s^{r}\right)}^{b-i}, \end{array}$$

where *s* is the Jaccard similarity of the two instances, *r* is the length of each band, and *b* is the number of bands. We can prove the derivative of *P*_new_(*s*) is greater than 0, which represents *P*_new_(*s*) a monotonically increasing function of *s*.

The number of false positive, denoted by FP_max⁡_(*k*), is

(5) $$\begin{array}{c} \text{F}{\text{P}}_{\max}\left(k\right)=\overset{J}{\underset{0}{\int }}N_{s}P_{\text{new}}\left(s\right)ds, \end{array}$$

where *N*_*s*_ denotes the total number of similar pairs whose Jaccard similarity is *s* in the instances set. Given an instance set, *N*_*s*_ is a constant. *J* is the given similarity threshold.

The value of FP_max⁡_(*k*) depends on the similarity distribution of a given instance set. The upper bound of FP_max⁡_(*k*) equals the original LSH FP_max⁡_(1). Without the knowledge of the similarity distribution of the data set, the lower bound of false positives cannot be directly derived. Hence, we introduce a threshold *ϵ* to ensure

(6) $$\begin{array}{c} \frac{\text{F}{\text{P}}_{\max}\left(k\right)}{\text{F}{\text{P}}_{\max}\left(1\right)}\leq \epsilon , \end{array}$$

where *ϵ* is close to zero with increasing *k*. If *k* is ⌊*Jn*/*r*⌋, the lower bound of false positives approximates to 0, which indicates that the candidates generated by the proposed new banding technique are almost all truly similar pairs.

To understand the zero lower bound with *k* = ⌊*Jn*/*r*⌋, suppose there are two signatures with *n* elements each, ⌊*Jn*/*r*⌋ bands of which are mapped to the same bucket. At least *r*⌊*Jn*/*r*⌋ ≈ *Jn* elements in the two signatures are identical because a band includes *r* elements. According to (2) and (3), the approximate similarity between the two corresponding instances is then greater than *Jn*/*n* = *J*. Hence, similarity for each pair of signatures is greater than the threshold *J* and no false positives exist.

**Figure 2:**
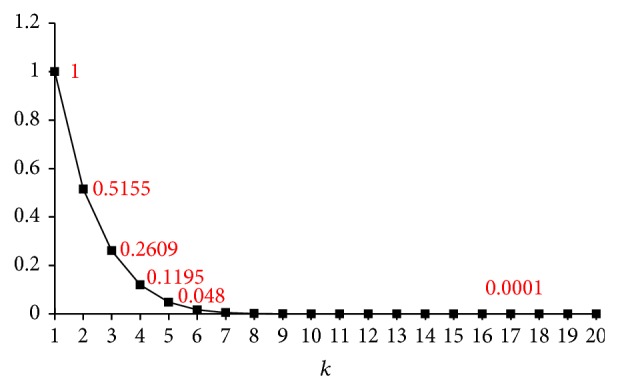
An illustrative example of number of false positives for various *k*.

The introduction of *ϵ* also enables us to personalize the number of false positives, that is, to vary the range of *k* for different *ϵ*. The range of *k* for a desired *ϵ* is a function of *J*, *b*, *r* that can be numerically solved. For example, given *J* = 0.7, *b* = 20, *r* = 5; Figure 2 shows the trend of FP_max⁡_(*k*)/FP_max⁡_(1) for *k*. The minimum of FP_max⁡_(*k*)/FP_max⁡_(1) is achieved when *k* = *b*. If the desired *ϵ* = 0.4, we can find a satisfying range of *k* ∈ [3,20] since FP_max⁡_(2)/FP_max⁡_(1) ≥ *ϵ* and FP_max⁡_(3)/FP_max⁡_(1) ≤ *ϵ*.

### 3.3. Number of False Negatives

False negatives are truly similar pairs mapped to disjoint bucket arrays. We also derive the upper and lower bound of false negatives generated by the proposed new banding technique.

**Lemma 2 .**

The upper bound of false negatives generated by the new banding technique is $\sum_{i=0}^{b-1}((\begin{array}{c} i\\ b \end{array}){\left(s^{r}\right)}^{i}{\left(1-s^{r}\right)}^{b-i})N_{s\geq J}$. The lower bound is close to the original LSH.

**Proof**

Similar to Section 3.2, the number of false negatives, denoted by FN_max⁡_(*k*), is

(7) $$\begin{array}{c} \text{F}{\text{N}}_{\max}\left(k\right)=\overset{1}{\underset{J}{\int }}N_{s}\left(1-P_{\text{new}}\left(s\right)\right)ds. \end{array}$$

FN_max⁡_(*k*) is a monotonic increasing function of *k*. The lower bound of it is achieved when *k* = 1. The upper bound of FN_max⁡_(*k*) is obtained when *k* is the total number of bands. Hence, the upper bound of FN_max⁡_(*k*) is proportional to the number of similar instances *N*_*s*≥*J*_:

(8) $$\begin{array}{c} \underset{k\to b}{\lim}\text{F}{\text{N}}_{\max}\left(k\right)=\left(\;\overset{b-1}{\underset{i=0}{\sum }}\left(\begin{array}{c} i\\ b \end{array}\right){\left(s^{r}\right)}^{i}{\left(1-s^{r}\right)}^{b-i}\right)N_{s\geq J}. \end{array}$$

**Figure 3:**
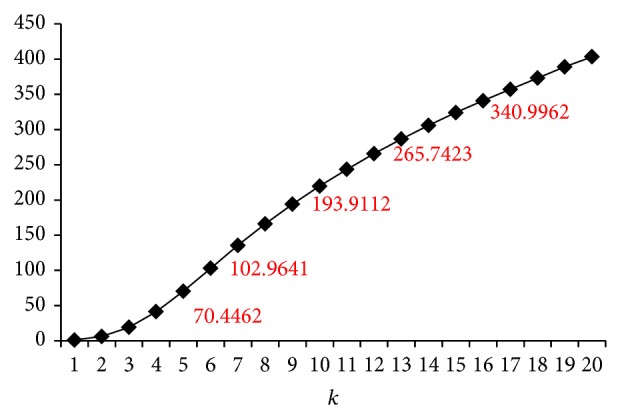
An illustrative example of number of false negatives for various *k*.

For a desired the number of false negatives, we do a division between FN_max⁡_(*k*) and FN_max⁡_(1) in terms of

(9) $$\begin{array}{c} \frac{\text{F}{\text{N}}_{\max}\left(k\right)}{\text{F}{\text{N}}_{\max}\left(1\right)}\leq \epsilon , \end{array}$$

where *ϵ* is a threshold which is always greater than 1. By deriving the numerical solution for $\int_{J}^{1}N_{s}(1-(\sum_{i=0}^{b-1}(\begin{array}{c} i\\ b \end{array}){\left(s^{r}\right)}^{i}{\left(1-s^{r}\right)}^{b-i}))ds$, the range of *k* for a desired *ϵ* is obtained. For example, given the arguments *J* = 0.7, *b* = 20, *r* = 5, Figure 3 shows us the trend of FN_max⁡_(*k*)/FN_max⁡_(1). If the desired *ϵ* = 100, from Figure 3, we can find that FN_max⁡_(5)/FN_max⁡_(1) ≈ 70 and FN_max⁡_(6)/FN_max⁡_(1) ≈ 100, so the satisfying range is *k* ∈ [1,5].

### 3.4. Balance False Positives and False Negatives

In some application scenarios, we want to have a balance between false positives and false negatives. Here we analyse a special case where we want a desired aggregated number of false positives and false negatives. We use FNP_max⁡_ to denote the sum of false positives and false negatives, which is defined as follows:

(10) $$\begin{array}{c} \text{FN}{\text{P}}_{\max}\left(k\right)=\text{F}{\text{P}}_{\max}\left(k\right)+\text{F}{\text{N}}_{\max}\left(k\right). \end{array}$$

The lower bound of FNP_max⁡_(*k*) is dependent on the similarity distribution of the given data set. However, since in most cases *N*_*s*<*J*_ ≫ *N*_*s*≥*J*_, thus FNP_max⁡_(*k*) = *N*_*s*<*J*_ *P*_new_(*k*) + *N*_*s*≫*J*_(1 − *P*_new_(*k*)) is less than FNP_max⁡_(1) when *k* is appropriately chosen.

**Figure 4:**
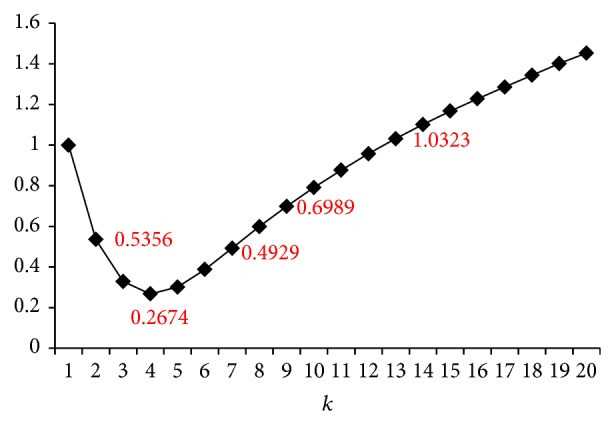
An illustrative example of balancing false positives and false negatives.

Inspired by Sections 3.2 and 3.3, we can also use a threshold *ϵ* to obtain the desired degree of precision. As shown in Figure 4, the ratio of FNP_max⁡_(*k*)/FNP_max⁡_(1) for *J* = 0.7, *b* = 20, *r* = 5 on a uniformly distributed data set first decreases as the value of *k* increases. The minimum is *ϵ* = 0.2674 when *k* = 4. Then the ratio increases as *k* becomes larger. If we are required to have a higher precision of the new banding technique, compared with traditional banding technique, in terms of aggregated number of false negatives and false positives (i.e., small FNP_max⁡_(*k*)/FNP_max⁡_(1) ≤ 1), then *k* ∈ [1,12] is acceptable.

## 4. MapReduce Implementation of PLSH

In this section, we first introduce the MapReduce framework. Then we present the details of implementing Personalized LSH with MapReduce, including minhashing, banding, and verification.

### 4.1. MapReduce

MapReduce [26] is a framework for processing paralleled algorithms on large scale data sets using a cluster of computers. MapReduce allows for distributed processing of data, which is partitioned and stored in a distributed file system (HDFS). Data is stored in the form of 〈*key*, *value*〉 pairs to facilitate computation.

**Figure 5:**
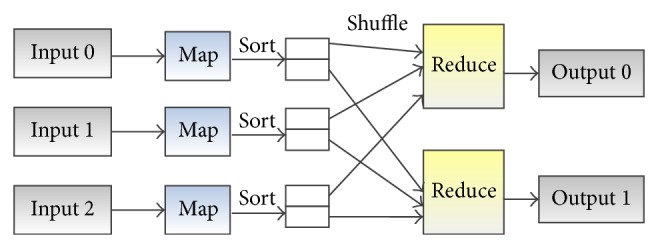
An illustrative example of MapReduce data flow, with 2 reduce tasks.

As illustrated in Figure 5, the MapReduce data flow consists of two key phases: the map phase and the reduce phase. In the map phase, each computing node works on the local input data and processes the input 〈*key*, *value*〉 pairs to a list of intermediate pairs 〈*key*, *value*〉 in a different domain. The 〈*key*, *value*〉 pairs generated in map phase are hash-partitioned and sorted by the key, and then they are sent across the computing cluster in a shuffle phase. In the reduce phase, pairs with the same key are passed to the same reduce task. User-provided functions are processed in the reduce task on each key to produce the desired output.

In similarity joins, to generate 〈*key*, *value*〉 pairs, we first segment the join attributes in each instance to tokens. Each token is denoted by a unique integer id. In the following steps, token id is used to represent each feature.

### 4.2. MinHashing

We use one map reduce job to implement minhashing. Before the map function is called, 〈*token*, *id*〉 pairs are loaded. In the map task, each instance is represented by a set of tokens {*id*} present in this instance.

In the reduce task, for each instance, the reducer produces a signature with length *n*. As described in Section 2, the minhashing function requires random permutations of features. But it is not feasible to permute massive features explicitly. Instead, a random hash function is adopted to simulate this task. Suppose the total number of features is *tc*, integer set *X* = [0,1,…, *tc* − 1], we choose the hash function as

(11) $$\begin{array}{c} h\left(i\right)=\left(a*i+b\right)\;\mathrm{mod}\;tc, \end{array}$$

where *a*, *b*, *i* ∈ [0, *tc* − 1] and *a*, *tc* must be relatively prime and *a* mod⁡ *b* is a function that obtains the remainder of *a* divided by *b*. It maps a number *i* ∈ [0, *tc* − 1] to another number *h*(*i*)∈[0, *tc* − 1] with no collision. Hence the result list *h*(0), *h*(1),…, *h*(*tc* − 1) is a permutation of the original features.

Furthermore, since it requires *n* independent permutations to produce a signature for each instance, we prove that there are more than *n* different permutations.

**Lemma 3 .**

Given *tc* features, the desired signature length *n*, the hash function *h*(*i*) = (*a*∗*i* + *b*) mod⁡ *tc*, where *a*, *b* and *i* ∈ [0, *tc* − 1], produces more than *n* different permutations.

**Proof**

Assume a permutation *x*_0_, *x*_1_,…, *x*_*tc*−1_ is generated by hash function *h* with parameters *a* and *b*; then *b* = *x*_0_, *x*_1_ = (*a* + *b*) mod⁡ *tc*, *a* = (*x*_1_ − *b* + *tc*)mod⁡ *tc*, and *x*_*k*+1_ = (*x*_*k*_ + *a*)mod⁡ *tc*, *k* ∈ [0, *tc* − 2]. Hence, for a specified *a*, different integers *b* ∈ [0, *tc* − 1] produce different permutations. Euler's totient function *ϕ*(*n*′) is an arithmetic function that counts the number of totatives of integer *n*′, which indicates the number of desired *a* is *ϕ*(*tc*). Therefore, there are *ϕ*(*tc*)*tc* pairs of 〈*a*, *b*〉 which produce *ϕ*(*tc*)*tc* different permutations. Since *ϕ*(*tc*)*tc* ≥ *tc* ≥ *n*, we prove that hash function *h* produces more than *n* different permutations.

### 4.3. Banding

**Figure 6:**
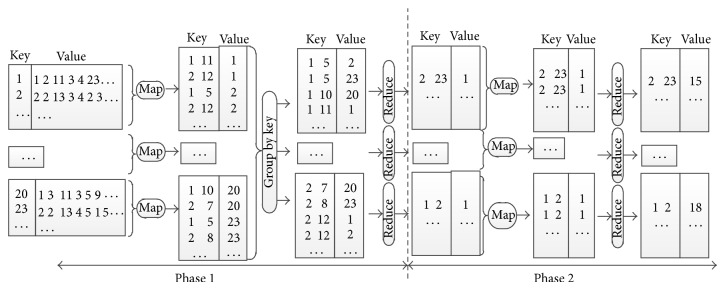
An illustrative example of data flow in banding stage.

Banding technique filters dissimilar pairs. As shown in Figure 6, we implement the banding procedure in two MapReduce phases.

In the first phase, the signatures are input to the map function. The map function divides each signature into *b* bands, each band consists of *r* elements, and then each band is mapped to a bucket. The outputs are in form of 〈[*bandId*, *bucketId*], *instanceId*〉. In other words, *bandId* and *bucketId* are combined as a key, and *instanceId* is assigned to the corresponding value. For example, as shown in Figure 6, the signature of instance 1 is (1 2 11 3 4 23 ⋯) and (2 2 13 3 4 23 ⋯) for instance 2. Suppose *r* = 3; then instance 1 is divided into at least 2 bands (1 2 11) and (3 4 23). The two bands are mapped to bucket 11 in bucket array 1 and bucket 12 in bucket array 2. So the outputs of map function for instance 1 include 〈[1,11], 1〉 and 〈[2,12], 1〉. Analogously, 〈[1,5], 2〉 and 〈[2,12], 2〉 are a part of map outputs for instance 2.

In reduce task, all instances with the same *bandId* and *bucketId* are assigned to the same reduce task. An output in the form of 〈[*instanceId*1, *instanceId*2], 1〉 is produced for every pair of instances, where the fixed value 1 represents the occurrence frequency for pair [*instanceId*1, *instanceId*2]. For instance, 〈[1,5], 2〉 and 〈[1,5], 23〉 are the aforementioned map outputs for instances 1 and 2, since they have the same *bandId* 1 and *bucketId* 5; the reduce task produces a pair 〈[2,23], 1〉. That is to say, instance 2 and instance 23 are likely to be a candidate pair because their first bands are both mapped to the 5th bucket.

In the second phase, the map task outputs what is produced in the first phrase. To minimize the network traffic between the map and reduce functions, we use a combine function to aggregate the outputs generated by the map function into partial local counts in the computing node. Subsequently, the reduce function computes the total counts for each instance pair. Outputs of the reduce function are in the form of 〈[*instanceId*1, *instanceId*2], *count*〉 pairs, where *count* is the global frequency for the instance pair. Personalized LSH eliminates those pairs of instances whose *count* is less than the given threshold *k*. As shown in Figure 6, suppose *k* = 12, the count of instance pair 〈[2,23], 15〉 is greater than *k*, so [2,23] is a candidate pair.

### 4.4. Verification

Candidate pairs generated in Section 4.3 need to be checked in the verification stage. For each candidate instance pair [*instanceId*1, *instanceId*2], signature similarity is computed. Because of the massive number of instances, it is not a trivial task.

**Figure 7:**
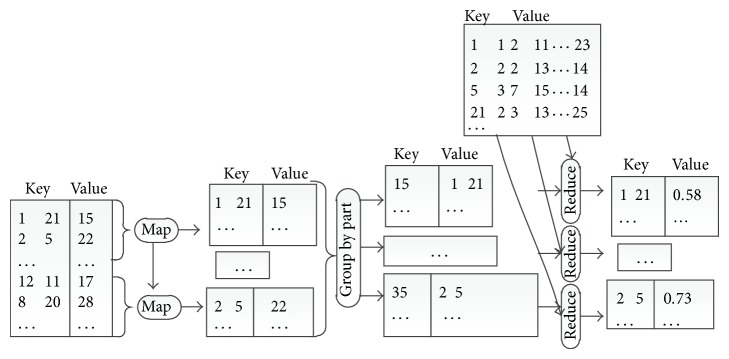
An illustrative example of the data flow in verification stage.

In order to reduce the storage cost for each reduce task, the set of signatures for all instances is partitioned into small files according to instance ids. In this way, each reduce task holds only two different small partitions, where the first partition is for the first instance in the pair [*instanceId*1, *instanceId*2], and the second partition is for the second instance. For example, for each reduce input pair [*instanceId*1, *instanceId*2], the first partition contains the signature for instance*Id*1 while the second partition contains the signature for *instanceId*2. All pairs of instances ids contained in the same partitions have to be assigned to the same reduce task. Hence, map task calculates the reduce task id for each pair according to its instances ids and produces an output 〈*reduceTaskId*, [*instanceId*1, *instanceId*2]〉. In reduce task, signatures similarity for each pair of instances is computed. Finally, reduce task outputs pairs whose similarities are greater than the given threshold *J*. The outputs are in the form of 〈[*instanceId*1, *instanceId*2], *sim*〉, where *sim* is the Jaccard similarity for *instanceId*1 and *instanceId*2. As shown in Figure 7, suppose the given threshold *J* = 0.7; similarity between signature 2 and signature 5 is 0.73, which is greater than *J*; the output is 〈[2,5], 0.73〉.

## 5. Experiment Evaluation

In this section, we design a series of experiments to analyze the performance of the proposed PLSH algorithms. We want to study the following research questions.

- The efficiency of PLSH: for example, is the PLSH fast, compared with other similarity join algorithms? Can it scale to large scale data sets? Will the different values of parameters affect the efficiency of PLSH?
- The effectiveness of PLSH: for example, is the PLSH accurate? Can it generate less false positives and false negatives?
- The personalization of PLSH: for example, how should we set the parameters of PLSH to generate the desired number of false positives and false negatives? Is the tailored PLSH more appropriate for different applications?

The experiments are conducted on a 6-node cluster. Each node has one processor i7-3820 3.6 GHz with four cores, 32 GB of RAM, and 100 G hard disks. On each node, we install the Ubuntu 12.04, 64-bit, server edition operating system, Java 1.6 with a 64-bit server JVM, and Hadoop 1.0. Apache Hadoop is an open source implementation of MapReduce. We run 2 reduce tasks in parallel on each node.

**Figure 8:**
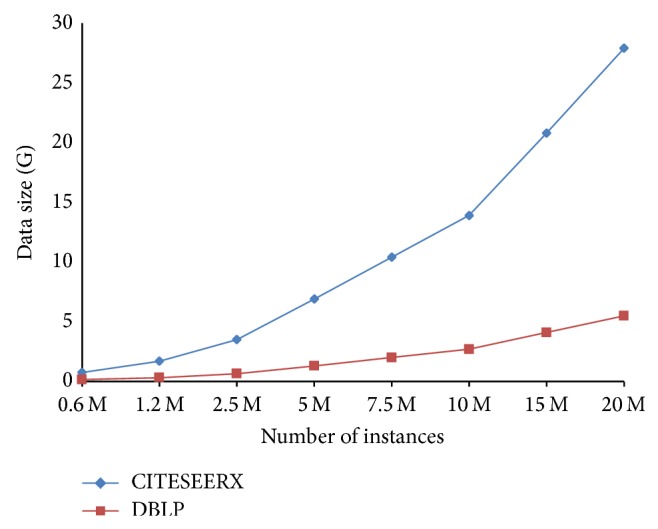
Dataset sizes of DBLP and CITESEERX.

We use DBLP and CITESEERX dataset and increase the number of instances when needed. The original DBLP dataset has approximately 1.2 M instances while the original CITESEERX has about 1.3 M instances. As shown in Figure 8, when increasing the number of instances of these two data sets, CITESEERX occupies larger storage than DBLP.

In our experiments, we tokenize the data by word. The concatenation of the paper title and the list of authors are the join attributes (i.e., paper title and the list of authors are two attributes in each instance.). The default threshold of Jaccard similarity *J* = 0.7. The default length of hash signature *n* is 100, and each band has *r* = 5 elements.

### 5.1. Efficiency of PLSH

The ground truth is the truly similar pairs generated by fuzzyjoin [12]. A 3-stage MapReduce approach is implemented for end-to-end set-similarity join algorithm and selects instance pairs with Jaccard similarities greater than the given threshold.

**Figure 9:**
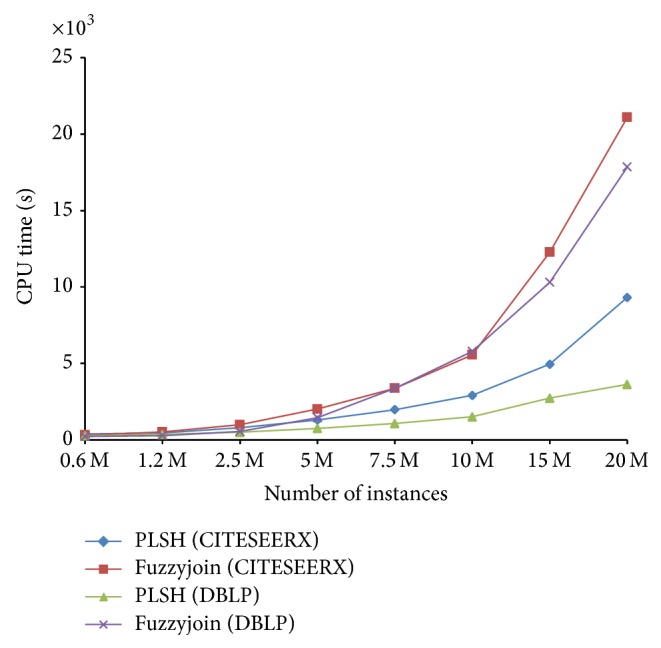
CPU time for various data sizes.

We first compare the efficiency of our method PLSH with fuzzyjoin on the DBLP and CITESEERX data sets. The CPU times of different algorithms are shown in Figure 9. We can conclude from Figure 9 that (1) generally PLSH is faster than fuzzyjoin. When the number of instances is 7.5 M in DBLP and CITESEERX, the time cost of fuzzyjoin is nearly two times of that of PLSH. (2) When the data size increases, the efficiency improvement is more significant. This suggests that PLSH is more scalable to large scale data sets. (3) Fuzzyjoin takes roughly equivalent CPU time on DBLP and CITESEERX with similar size, while PLSH works faster on DBLP than on CITESEERX. This suggests that PLSH is more affected by the similarity distribution in a data set.

Next we analyze the effect of the number of reduce tasks for algorithm efficiency. Because the reduce task number of verification step is different from the previous steps, we record the CPU time in the previous three stages (minhashing, banding1, and banding2, note that banding procedure is completed in two MapReduce phases) and in verification step separately. In the three steps, we vary the reduce tasks number from 2 to 12 with a step size of 2. The data sets are DBLP datasets containing 0.6 M, 1.2 M, 2.5 M, 5 M, and 7.5 M instances, respectively.

**Figure 10:**
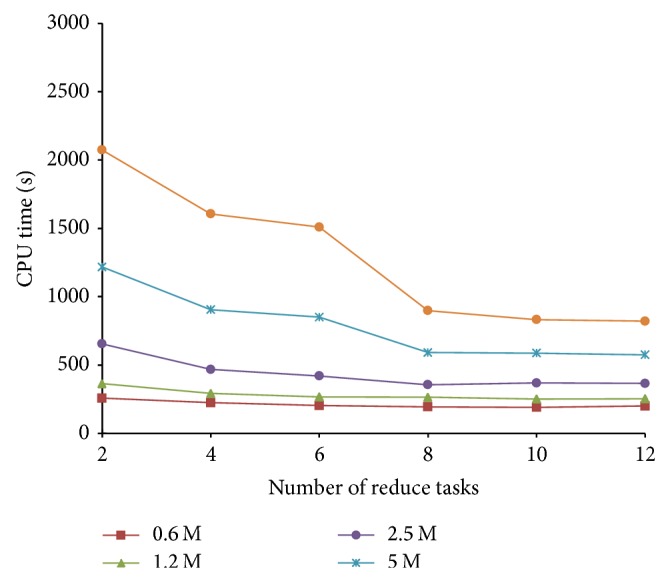
CPU time of stages previous to verification for different data sizes with various reduce task number.

From Figure 10 we have the following observations. (1) In general, the time cost of PLSH reduces with more reduce tasks. This verifies our assumption that the parallel mechanism improves the algorithm efficiency. (2) When there are less than 2.5 M instances, CPU time decreases slowly with the increasing reduce tasks number. This is because the start time of MapReduce cannot be shortened even though tasks number is increasing. When the CPU time is more than 2000 seconds, the percentage of start time is just a small part of the total time. Therefore, when the number of instances is relatively large, the improvement is more significant. This suggests that the parallel mechanism of PLSH is more suitable for large scale data sets.

**Figure 11:**
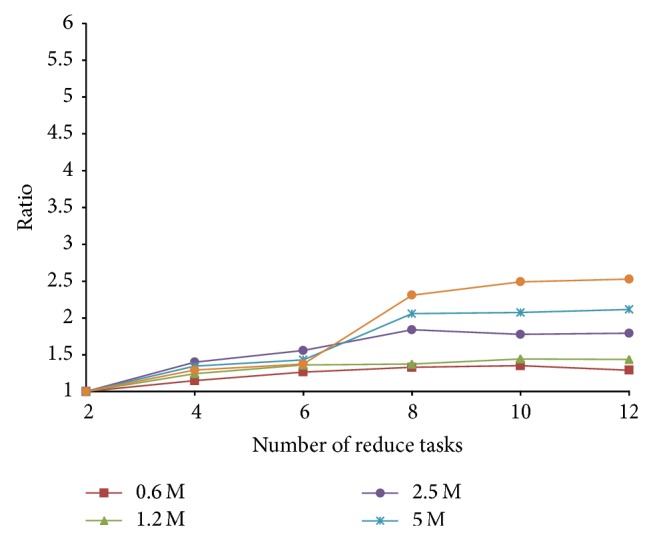
Relative CPU time for different data sizes with various reduce task number.

We further study the speedup of reduce task numbers. We set the time cost with 2 reduce tasks as the baseline and plot the ratio between the running time versus the baseline for various reduce task numbers. From Figure 11, we observe that when the size of dataset increases, the speedup is more significant. But none of the curves is a straight line, which suggest that the speedup is not linear with respect to the number of reduce tasks. The limited speedup is due to two main reasons. (1) There are 5 jobs to find the similar instances. The start time of each job will not be shortened. (2) As the number of dataset increases, more data is sent through the network, and more data is merged and reduced; thus the traffic cost cannot be reduced.

**Figure 12:**
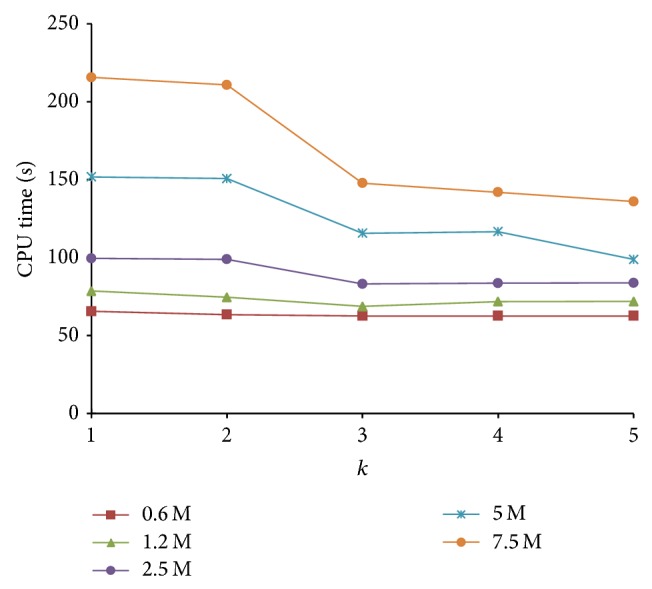
Verification time with various *k*.

The number of reduce tasks is fixed in the verification stage. However, in this stage, parameter *k* in the proposed PLSH has a significant effect on the running time. We analyze the performance of PLSH with various values of *k*. From Figure 12, we have the following observations. (1) In general, as the value of *k* increases, the time cost in verification stage decreases. This suggests that, with a larger *k*, the proposed PLSH can better prune dissimilar pairs and enhance efficiency. (2) When the data set is small, that is, the number of instances is smaller than 2.5 M, the enhancement is not obvious. The underlying reason is that there are fewer candidates in a small data set. (3) Speedup is significant in large scale data set (with more than 5 M instances), which suggests the scalability of PLSH.

### 5.2. Effectiveness Evaluation

In this subsection, we study the effectiveness of PLSH, and how the length of signature, the defined threshold, and the parameter *k* affect effectiveness.

We first define four types of instances. False positives (FP) are dissimilar pairs (similarity lower than the threshold) that are selected as candidates by PLSH. True positives (TP) are similar pairs (similarity higher than the threshold) that are selected as candidates by PLSH. False negatives (FN) are similar pairs (similarity higher than the threshold) that are pruned by PLSH. True Negatives (TN) are dissimilar pairs (similarity lower than the threshold) that are pruned by PLSH.

**Figure 13:**
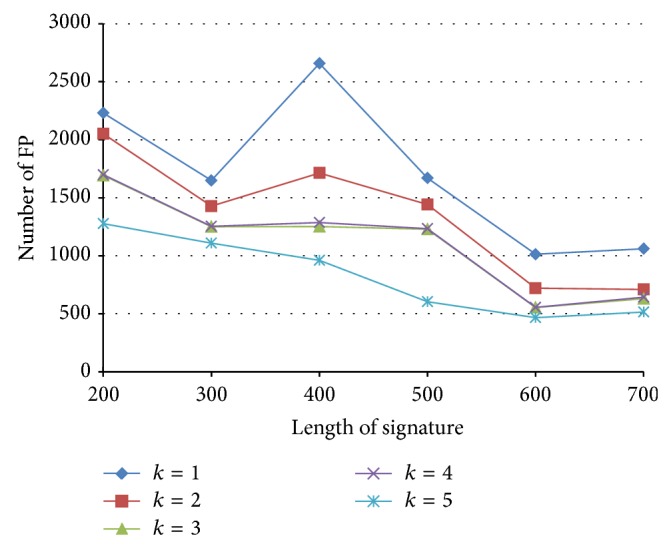
The number of false positives with various signature length and *k*.

The numbers of FP with various signature length and *k*, with similarity threshold *J* = 0.7, are shown in Figure 13. We have the following observations. (1) For a fixed signature length, the number of false positives significantly decreases with larger *k*. (2) For larger *k* (e.g., *k* = 5), prolonging the signature results in fewer false positives. (3) For small *k* and shorter signatures (<400 elements), the number of false positives is uncertain. The above three observations indicate that the proposed PLSH can achieve a high precision with larger *k*, longer signatures.

**Figure 14:**
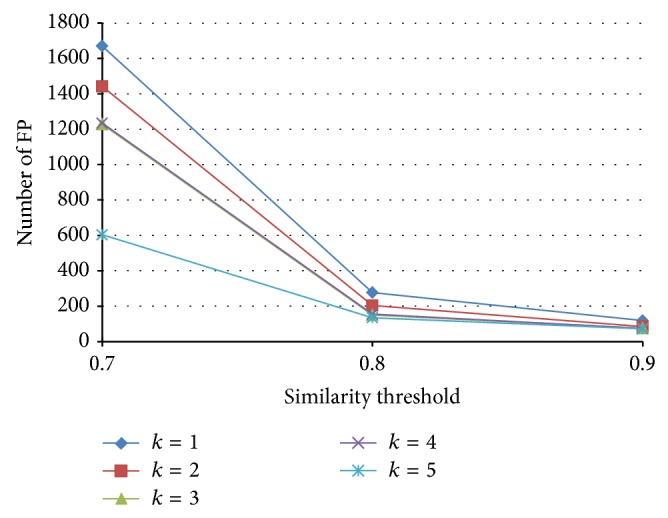
Number of false positives with various similarity thresholds and *k*.

The numbers of FP with various similarity threshold and *k*, with signature length *n* = 500, are shown in Figure 14. We have the following observations. (1) For a fixed similarity threshold, the number of false positives significantly decreases with larger *k*. (2) For a fixed *k*, the number of false positives significantly decreases with larger similarity threshold. This is because, with larger similarity threshold, there are fewer qualified candidates.

**Figure 15:**
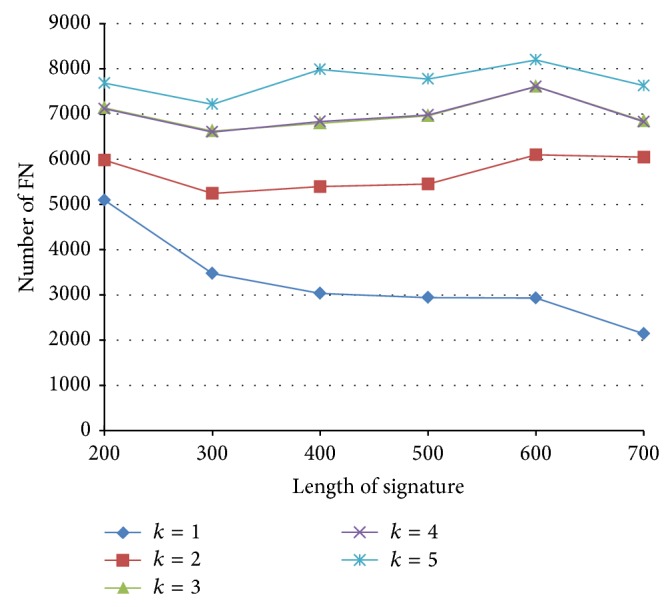
The number of false negatives with various signature lengths and *k*.

The numbers of FN with various signature length and *k*, with similarity threshold *J* = 0.7, are shown in Figure 15. We have the following observations. (1) For a fixed signature length, the number of false negatives generated by original LSH (*k* = 1) is fewer than PLSH. (2) For original LSH, shorter signatures generate more FN. (3) In terms of FN, PLSH is not very sensitive to the length of signature.

**Figure 16:**
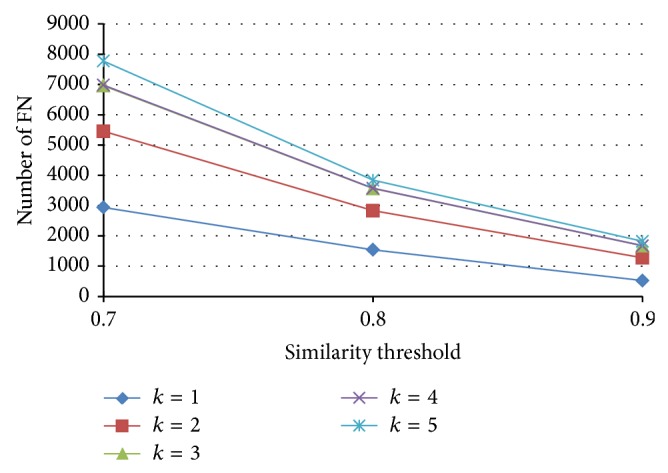
The number of false negatives with various similarity thresholds and *k*.

The numbers of FN with various similarity threshold and *k*, with signature length *n* = 500, are shown in Figure 16. Although the number of false negatives for PLSH is larger than the original PLSH, we observe that for large similarity threshold *J* = 0.9 the difference is minute. In most applications, we want to search for very similar pairs; thus, the PLSH will still perform good in most scenarios.

Since the false negatives generated by PLSH are in general more than LSH, we further use precision and specificity to evaluate the performance of PLSH. Precision is defined as PR = TP/(TP + FP) and specificity is defined as TN/(FP + TN). In this subsection, we want to analyze the effect of *k*, *n*, and *J* in terms of precision and specificity. We vary *k* from 1 to 5 with a step size of 1, *n* from 200 to 500 with a step size 100, and *J* from 0.7 to 0.9 with a step size 0.1.

**Figure 17:**
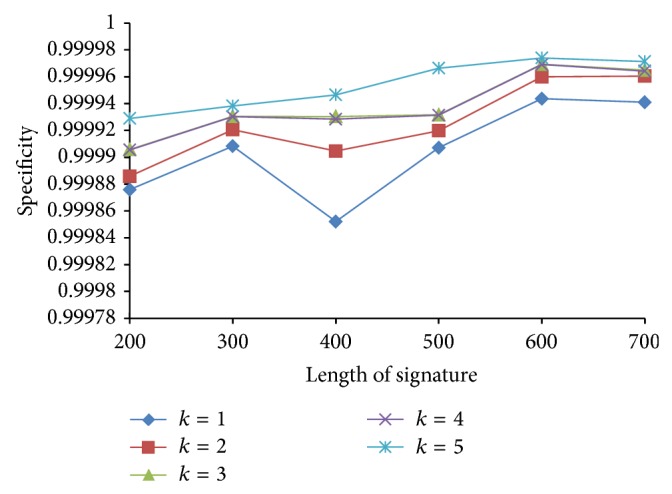
Specificity with various *n* and *k*.

**Figure 18 fig18:**
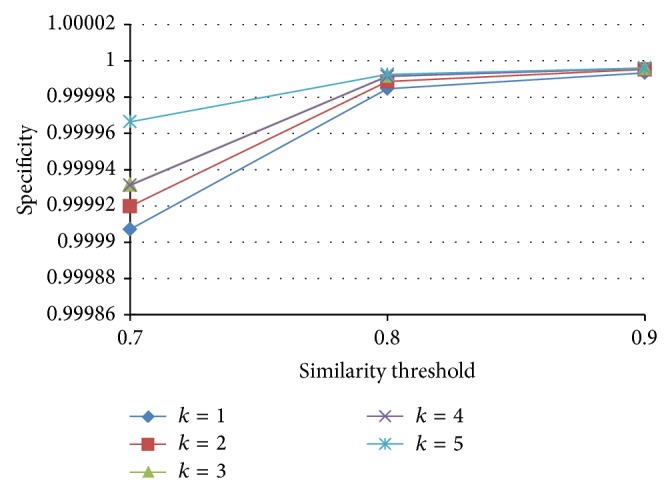
Specificity with various *J* and *k*.

**Figure 19 fig19:**
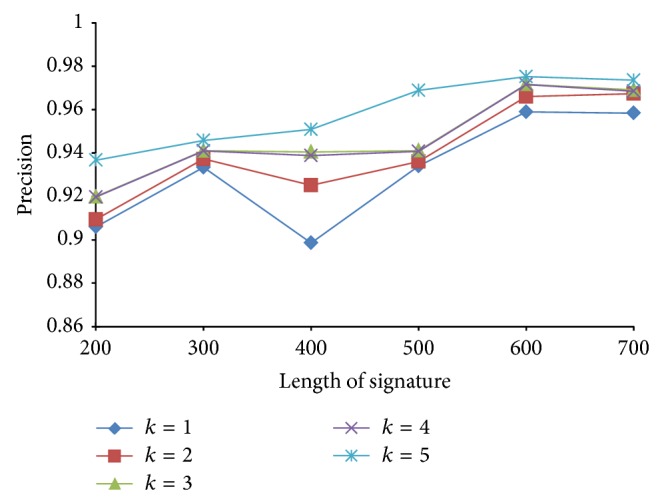
Precision with various *n* and *k*.

**Figure 20:**
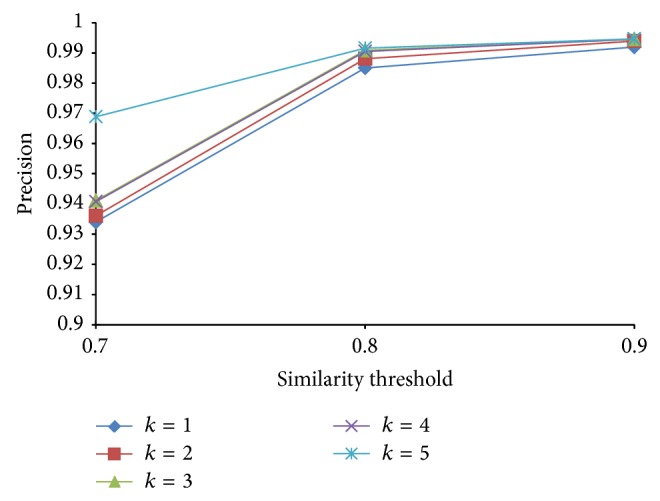
Precision with various *J* and *k*.

From Figures 17 [Fig fig18] [Fig fig19] to 20, we have the following observations. (1) The PLSH method performs better than LSH in terms of specificity and precision, which demonstrates the potential of PLSH in many data mining and bioinformatics applications. (2) Generally, longer signatures outperform shorter signatures.

### 5.3. Personalized LSH

As proved in Section 3, an important characteristic of our proposed PLSH is that it is capable of tailoring the number of false positives and false negatives for different applications. In this subsection, we present a pilot study on the tailoring capability of PLSH. We first numerically derive the appropriate *k* for different degree of desired precision. As shown in Table 3, the required precision is measured in terms of the ratio of false positives versus conventional LSH FP_max⁡_(*k*)/FP_max⁡_(1), the ratio of false negatives versus conventional LSH FN_max⁡_(*k*)/FN_max⁡_(1), and the ratio of total errors (the sum of false positives and false negatives) versus the conventional LSH FNP_max⁡_(*k*)/FNP_max⁡_(1). For example, we can see that if we want to generate less than half of false negatives in LSH, FN_max⁡_(*k*)/FN_max⁡_(1) ≤ 0.5, we should set *k* = 3.

We then use the different settings of *k* to generate collaborator recommendations on DBLP and CITESEERX data sets. We keep the authors who have published more than 25 papers. We use a modified collaborative filtering [25] to generate recommendations. For each paper *p*_*a*_ published by author *a*, PLSH is utilized to find a set of similar publications {*r*} with similarity sim(*r*, *p*_*a*_) ≥ *J*,*J* = 0.7. We then gather the set of authors {*a*(*r*)} who write the similar publications. In recommendation systems, the similar authors are treated as nearest neighbors. Each nearest neighbor is assigned a score, which is the accumulated similarities between the publication of nearest neighbor and the publication of the author *a*. For each author, the top 5 nearest neighbors with largest accumulated similarities are returned as recommended collaborators. We manually annotate the results. The evaluation metric is the precision at top 5 results (*P*@5). The average *P*@5 results are shown in Table 3. We have the following conclusions. (1) When the number of false positives is reduced, we can generate more precise nearest neighbours; thus the recommendation performance is boosted. (2) When the number of false positives is too small, then due to the sparsity problem, the collaborative filtering framework is not able to generate enough nearest neighbors; thus the recommendation performance is deteriorated. (3) A recommendation system achieves best performance with a fine tuned parameter *k*, which optimizes the trade-off between false positives and false negatives.

## 6. Related Work

There are fruitful research works on the similarity joins problem. Commonly adopted similarity measurements include cosine similarity [7], edit distance [8], Jaccard similarity [12], hamming distance [10], and synonym based similarity [27].

The methodologies used in similarity joins can be categorized into two classes. The first class adopts dimension reduction technologies [16], so that the similarity computation is conducted in a lower dimensional space. The second class utilizes filtering approaches to avoid unnecessary similarity computation, including prefix filter [7], length filter [7], prefix filter [20] and suffix filter [21], and positional filter [8]. To take advantage of the two categories, a series of hashing methods (such as minhashing [17], minwise hashing [18], and Locality Sensitive Hashing (LSH) [10, 19]) combine dimension reduction and filtering approaches by first using a signature scheme [28] to project the original feature vectors to low dimensional signatures and then filter unqualified signatures.

Recently, parallel algorithms are adopted on clusters of machines to improve efficiency of similarity joins for large scale and high dimensional data sets. We have seen an emerging trend of research efforts in this line, including a 3-stage MapReduce approach for end-to-end set-similarity join algorithm [12, 13], fast computation of inner products for large scale news articles [13], a new ant colony optimization algorithm parallelized using MapReduce [14] to select features in a high dimension space, and the LSS algorithm exploiting the high data throughput of GPU [15].

In the era of big data, approximate computation is also a possible solution, if the approximate result with much less computation cost is pretty close to the accurate solution. Approximate computation can be implemented not only through hardware modules [29, 30] but also through approximate algorithms [31, 32]. Unlike exact similarity joins [25], approximate similarity joins and top-*k* similarity joins [33] are better adapted to different application domains [34].

## 7. Conclusion

The problem of similarity joins based on Jaccard similarity is studied. We propose a new banding technique which reduces the number of false positives, false negatives, and the sum of them. From our experiments, it is shown that our new method is both efficient and effective.

## Figures and Tables

**Table 1:** An illustrative example of similarity joins based on Jaccard similarity. 0/1 indicates absence/presence of features in each instance.

Instance	Feature					
	*a*	*b*	*c*	*d*	*e*	*f*
*A *	0	1	0	0	1	0
*B *	1	0	0	0	1	1
*C *	0	1	0	1	1	0
*D *	0	0	1	1	0	0
*E *	0	0	0	1	0	1

**Table 2:** An illustrative example of permutation of feature vectors. 0/1 indicates absence/presence of features in each instance.

Instance	Feature					
	*b *	*a *	*c *	*d *	*f *	*e *
*A *	**1**	0	0	0	0	1
*B *	0	**1**	0	0	1	1
*C *	**1**	0	0	1	0	1
*D *	0	0	**1**	1	0	0
*E *	0	0	0	**1**	1	0

**Table 3:** Recommendation performance with different *k*.

*k*	FP_max⁡_(*k*)/FP_max⁡_(1)	FN_max⁡_(*k*)/FN_max⁡_(1)	FNP_max⁡_(*k*)/FNP_max⁡_(1)	*P*@5DBLP	*P*@5CITESEERX
1	1	7	1	0.71	0.82
2	0.9	20	0.9	0.83	0.90
3	0.5	42	0.5	0.91	0.99
4	0.2	71	0.3	0.99	0.93
5	0.1	103	0.4	0.97	0.84
